# Mindfulness-based interventions for children and adolescents across all settings: a scoping review protocol

**DOI:** 10.1186/s13643-020-01548-7

**Published:** 2020-12-07

**Authors:** Marie-France Perrier, Nalia Gurgel-Juarez, Heather Leslie Flowers, Anna McCormick, Sarah J. Short

**Affiliations:** 1 School of Rehabilitation Sciences, Faculty of Health Sciences, University of Ottawa, Ottawa, ON Canada; 2 Children’s Hospital of Eastern Ontario, Ottawa, ON Canada; 3 Ottawa Hospital Research Institute, Ottawa, ON Canada; 4 Institut du savoir - A Knowledge Institute, Montfort Hospital, Ottawa, ON Canada; 5 Department of Pediatrics, Faculty of Medicine, University of Ottawa, Ottawa, ON Canada; 6 Department of Educational Psychology, School of Education, University of Wisconsin–Madison, Madison, WI USA; 7 Center for Healthy Minds, University of Wisconsin–Madison, Madison, WI USA

**Keywords:** Children, Adolescents, Pediatric, Mindfulness, Meditation, Mindfulness-based interventions, Scoping review

## Abstract

**Background and purpose:**

Although mindfulness-based interventions (MBIs) are becoming increasingly popular, the application of MBIs with children and adolescents is still in its infancy. Mapping the existing literature is necessary to help guide pediatric mindfulness interventions. Our purpose is to synthesize the evidence of reported MBIs for children and adolescents with and without physical, mental, and cognitive disorders. Accordingly, we aim to identify trends and gaps in the literature, so that we can provide direction to researchers who seek to advance the evidence base for using MBIs in pediatric populations.

**Methods:**

Our search strategy will be conducted following Arksey and O’Malley’s methodological framework. It will include a comprehensive search of published studies in 7 databases, gray literature, conference proceedings, and citations of selected articles. Two independent reviewers will evaluate all abstracts and full articles that have a pediatric sample (children 2–17 years), use MBIs to promote development or to remediate underlying disorders, and are written in English or French. We will identify the definitions and concepts from MBIs, categorize accepted studies according to etiology and rehabilitation type, describe intervention methodology, and report outcomes of selected studies.

**Discussion:**

Our review will provide a comprehensive overview of the pediatric mindfulness intervention literature to date, involving a range of mental, cognitive, and physical outcomes for healthy children and adolescents and for those with a variety of disorders in clinical and institutional settings. We will disseminate results to mindfulness practitioners and provide guidance to future pediatric researchers in their development and application of mindfulness interventions, thereby contributing to the scientific understanding of mindfulness for the ultimate betterment of child and adolescent well-being and life-long functioning.

**Systematic review registration:**

PROSPERO does not accept scoping review protocols.

**Supplementary Information:**

The online version contains supplementary material available at 10.1186/s13643-020-01548-7.

## Background

### Rationale

The practice of mindfulness meditation has gained popularity in recent decades alongside a surge of academic journal publications. In fact, the notion of mindfulness has become mainstream and perhaps even popularized in recent years. The American Mindfulness Research Association (AMRA) reports a steady yearly growth in the number of published papers involving the word “mindfulness” in the title (ranging from 10 in the year 2000 to 842 in 2018 in the Web of Science database) [1]. The integration of mindfulness-based interventions (MBIs) with traditional therapies is also increasing in a variety of clinical and institutional settings [2, 3], but almost exclusively in adult populations. Only recently has the focus shifted toward the application of MBIs with children and adolescents [4].

Mindfulness is the practice of directing attention in a particular way. It involves “awareness that emerges through paying attention on purpose, in the present moment, and nonjudgmentally” ([3], p145), which can be cultivated and developed through meditation practice. In medicine and psychology, MBIs have been associated with better maintenance of therapeutic gains relating to depression, anxiety, and pain [2]. By extension, MBIs can arguably improve physical health conditions, cognitive, psychosocial, and affective outcomes [2, 4–11]. Because of the applicability of mindfulness to many areas of functioning, there is increasing interest in its application. To this end, mindfulness interventions could be desirable and advantageous starting in early childhood to promote healthy development or to forestall anticipated setbacks or mitigate the effects of existing illnesses or deficits.

In fact, the evidence base is growing with respect to demonstrating improved mental and physical health in adults [2, 7, 12, 13]. In Khoury and his colleagues’ meta-analysis of mindfulness-based therapy for a range of physical and psychological conditions, mindfulness interventions were found to be moderately effective in pre-post designs (Hedge’s *g* = .55) and in comparative designs (with waitlist controls) (Hedge’s *g* = .53) [12]. Among the afore-mentioned designs, MBIs showed large and clinically significant effects in treating anxiety and depression. When MBIs were compared with other active treatments (e.g., psychoeducation, relaxation, imagery), smaller effect sizes resulted (Hedge’s *g* = .33). Similarly, effect sizes for primary psychological outcomes (anxiety, depression, and stress) were moderate (Hedge’s *g* ≥ 5) in another recent meta-analysis on mindfulness-based stress reduction (MBSR) randomized controlled trials (RCTs) [13] for designs with waitlists and treatment as usual controls.

Emerging mindfulness research shows a preliminary parallel in pediatric mental health and cognitive outcomes as well as an upsurge of adapted MBIs for children [4–7, 14, 15]. Adapted mindfulness interventions are those that have been modified from adult interventions to ensure developmental appropriateness for children and adolescent populations. Examples of adaptations include using age-appropriate language and practices that fit the child’s developmental stage or needs (e.g., attention span or cognitive abilities); shortening intervention sessions and individual training; modifying or omitting home mindfulness practice in favor of stories, games, and activities (e.g., drawing, listening to music, tasting and smelling foods); and incorporating more extended family involvement [5, 8, 10, 12, 15, 16]. Childhood and adolescence may in fact be an ideal developmental period to introduce mindfulness to enhance cognitive development, particularly with respect to executive functions and self-regulation skills [17, 18].

Due to the growing interest in pediatric mindfulness interventions, experts urge further research involving children to ensure the development of well-suited, justifiable, and adapted mindfulness interventions [2]. Emerging research conducted with children and adolescent populations demonstrates promise. Multiple reviews have synthesized the evidence for the feasibility and acceptability of MBIs [5, 11, 15, 16]. Additionally, there is emerging evidence for improvements in cognitive and psychosocial outcomes following MBIs in child and adolescent populations, as well as an overall increase in well-being [2, 8, 9, 14–16, 18, 19].

A recent meta-analysis of RCTs [4] undertaken in children and adolescents demonstrated small effect sizes (Cohen’s *d* > .2 to *d* < .5) for MBIs relative to controls for various outcomes of interest (mindfulness, executive functioning, attention, depression, anxiety/stress, and negative behaviors). Individual studies have reported overall small positive treatment effects for experimental and quasi-experimental designs when MBIs were applied for treating psychological outcomes (cognitive performance, stress, and resilience) and across a variety of therapeutic outcome domains (academic achievement and school functioning, emotional/behavioral regulation, meta-cognition and cognitive flexibility, and physical health) [10, 14].

Other research syntheses point to moderate effects for MBSR in reducing depressive symptoms relative to control groups (no treatment, treatment as usual, or active control) [9, 13]. To illustrate, Pandey and her colleagues [18] found a small effect size favoring mindfulness/yoga intervention groups for mental health and academic achievement. Saltzman and Goldin [16] also support the use of an adapted MBSR by reporting significant improvements and clinical change in attention, emotional reactivity, and meta-cognitive functioning, compared to families on the waitlist.

Renshaw and Cook [20] describe an increased interest in the use of MBIs in youth and in school and clinical settings. The authors delineate an agenda for future mindfulness research in schools relative to cognitive gains. Other reviews have reported on cognitive (such as executive functions, attention, self-regulation, and behavior) [2, 5, 7, 8, 10, 14] and psychosocial (such as prosocial attitudes, emotional regulation, stress, anxiety, and depression symptom reduction) outcomes [2, 4–11, 14, 15] following MBIs, which in turn may improve academic scores and cognitive performance in clinical and institutional settings [4–6, 8, 10, 14, 18]. Studies investigating physiological biomarkers (such as blood pressure and heart rate) [6, 8, 14] and physical symptoms (such as reflux symptoms, body weight, pain acceptance, and sleep quality) [5, 7, 11, 14] also report promising outcomes of postintervention improvements. Taken together, the existing literature is beginning to show promise for the effectiveness of adapted mindfulness interventions in pediatric populations.

Notwithstanding, there remains a need to continue to corroborate the effects of MBIs in children and adolescents and to establish reproducible interventions. A crucial first step involves summarizing and disseminating research patterns and identifying gaps in the evidence base. We anticipate that our scoping review will provide guidance to clinicians on how to offer MBIs according to current applications from the evidence, as well as direct future pediatric researchers in their development of age-appropriate and sustainable mindfulness interventions.

### Objective

The primary purpose of our scoping review is to identify the breadth of literature reporting MBIs for both healthy children and adolescents and those with physical, mental, and cognitive disorders. The resulting review may inform practitioners and researchers working with MBIs.

## Methods

A scoping review is the most suitable type of review for synthesizing a potentially broad and diverse literature base on MBI use in children and adolescents. Our review will provide an overview of existing evidence for MBI research, including its extent, range, and nature in the realm of pediatric interventions [19].

We will conduct our scoping review according to the methodological framework proposed by Arksey and O’Malley [21]. The steps involved are (1) identifying the research question; (2) identifying relevant studies; (3) selecting studies for inclusion; (4) charting the data; (5) collating, summarizing, and reporting the results; and (6) undertaking an optional consultation exercise.

### Research question and objective

The application of mindfulness is rapidly growing, yet mindfulness research primarily involves adult populations [7]. We aim to map the existing literature to summarize the overall state of research activity involving MBIs for children and adolescents. This scoping review then aims to answer the following research question: What mindfulness-based interventions have been developed and used in childhood and adolescence?

Our overarching objective is to identify the breadth of literature reporting MBIs for both healthy children and adolescents and those with physical, mental, and cognitive disorders. Specifically, we intend to map emerging trends in pediatric research applying MBIs in both healthy populations and those with congenital or acquired disorders [2]. Accordingly, we will identify and synthesize the various definitions and concepts that have been used in MBIs, determine the etiology and/or rehabilitation type MBIs have been used with, describe the methodology and characteristics of MBIs, and determine the outcomes of the selected studies. We recognize that revisions to our objective may be necessary as we engage in an iterative process to gain a thorough understanding of the subject matter throughout the course of our review [22].

### Identifying relevant studies

#### Eligibility criteria

We will include studies based on the Participants–Concept–Context (PCC) mnemonic recommended by the Joanna Briggs Institute (JBI) for scoping reviews [23]. Participants will include children and adolescents from 2 to 17 years of age. The lower end of the age range was determined by database age limits, which include 2-year-olds in the preschool range (2– years of age). The review will involve MBIs conducted in healthy children and adolescents and those with physical, mental, and cognitive disorders, whether congenital or acquired. There will be no restrictions to modes of delivery, intervention type or duration, or health care provider background. The context where the MBIs occurred will not be restricted to any geographical location or setting. Consequently, studies conducted in institutional, clinical, or home settings from any location will be eligible.

We will exclude articles involving interventions that are not based on mindfulness practices (as defined herein). For the purposes of this scoping review, mindfulness will include the practice of directing attention in a particular way involving “awareness that emerges through paying attention on purpose, in the present moment, and nonjudgmentally” ([3], p145). As such, mindfulness intervention does not refer to relaxation, thought suppression, mood management techniques, or meditation alone [24]. Additionally, we will consider Bishop and his colleagues’ two-component operational definition describing mindfulness as “the self-regulation of attention so that it is maintained on immediate experience, thereby allowing for increased recognition of mental events in the present moment” ([24], p232), and “the adoption of a particular orientation toward one’s experiences in the present moment, an orientation that is characterized by curiosity, openness, and acceptance” ([24], p232).

#### Search strategy

We will include studies available in English or French language from a variety of electronic sources to ensure the comprehensiveness of our scoping review. Accordingly, we will search the following databases: CINAHL, Embase, ERIC, MEDLINE, PsycInfo, AMED, and Scopus from the first date of their online availability. Search terms will include Medical Subject Headings (MeSH) and context-dependent terms (e.g., title, abstract, and keywords) related to the concepts of this study. The search strategy for MEDLINE is provided in Table 1. We will apply corresponding search terms to the other six databases.

**Table 1:** MEDLINE search strategy (1946 to present)

1. exp Child/	
2. adolescent/	
3. (pediatric* or paediatric* or child* or preschool* or pre-school* or kindergarten* or kindergarden* orelementary school* or nursery school* or schoolchild* or toddler* or boy or boys or girl* or middle school*or pubescen* or juvenile* or teen* or youth* or high school* or adolesc* or pre-pubesc* or prepubesc*).ti,ab,kf.	
4. or/1-3	
5. mindfulness/	
6. meditation/	
7. mindful*.ti,ab,kf.	
8. meditat*.ti,ab,kf.	
9. (Mindfulness-based stress reduction).ti,ab,kf.	
10. (Mindfulness-based cognitive).ti,ab,kf.	
11. MBSR.ti,ab,kf.	
12. MBCT.ti,ab,kf.	
13. or/5-12	
14. and/4,13	

In addition to databases, we will search proceedings of relevant international conferences, as well as the gray literature (see Table 2). For all accepted studies, we will review reference lists and citations through Google Scholar using a process of forward and backward chaining [25].

**Table 2:** List of search sources including databases, conference proceedings, and gray literature

Literature type	Sources
Databases	CINHAL Embase ERIC MEDLINE PsycInfo AMED Scopus
Conference proceedings	World Pediatrics Society for Research in Child Development International Symposium for Contemplative Research Mindfulness Research Conference – Mindful Families Schools & Communities
Gray literature	Open Grey Grey Matters Networked Digital Library of Theses and Dissertations Open Access Theses and Dissertations Proquest Dissertations and Theses Global Ontario Public Health Libraries Association

### Selecting studies for inclusion

We will use a three-step process to select the studies for this scoping review: (1) extracting search results and removing duplicates, (2) title and abstract review, and (3) full article review. We will use Microsoft Excel and Covidence software [26] throughout the process to support and streamline the production of this scoping review. After eliminating duplicates and citations without abstracts, two independent reviewers will review all titles and abstracts, coding them for acceptance or exclusion according to the inclusion criteria.

In the final step, we will retrieve all potentially relevant papers and conduct independent evaluation by two reviewers, again using pre-established coding criteria. To facilitate decisions for the full article review process, study authors will be contacted for further information if necessary. At both the title and abstract (step 2) and full article review (step 3) stages, discrepancies will be resolved by discussion and consensus between the two reviewers. If they fail to reach consensus, a third reviewer will then review the article and discuss further to permit a final decision. We will document the reasons for the exclusion of full articles and report associated frequencies in our scoping review. We have designed our scoping review according to the PRISMA-ScR where applicable (see Additional file 1).

### Charting the data

Based on the final set of accepted articles, we will collect the data using a charting approach followed by data verification. The data will be sorted according to areas of interest (see Table 3) from the included studies such as publication specifics (date and location of study), population, setting, design, intervention type, and key findings relevant to the review objective.

**Table 3:** Data extraction table

Categories	Characteristics
1. Article details	Year of publication Location of study (country)
2. Study details	Design Aims/purpose Setting Population
3. Sample characteristics	Sample size Groups Age range Sex/gender Race/ethnicity Socio-economic status Language(s) used Primary etiology (if any) Medical history
4. Intervention details	Name Type of MBI Subtypes of MBI Adaptations Interventionist/facilitator Formal personal mindfulness practice Professional training Instructor qualification/training Formal teaching experience Health care professional Caregiver Individual participants (self-administered) Length Frequency (sessions, training program) Duration Location/context/practice setting International Classification of Functioning, Disability, and Health (ICF) codes (body functions, activities, and participation) Target audience
5. Outcomes	Barriers or facilitators Outcome measures/tools Domains of intervention use

### Collating, summarizing, and reporting the results

After gathering and examining the evidence, we will summarize data in a tabular form (using data and charts) for categories such as publication trends, study design, and intervention type. We will provide a narrative summary where relevant (e.g., for MBI concepts and definitions). Given our specific objective of mapping research in pediatric MBIs, we will organize the literature thematically under main conceptual categories. Types of MBIs for children and adolescents will be categorized according to etiology and rehabilitation type where applicable, since we are interested in mindfulness interventions to promote development or to remediate underlying disorders.

### Optional consultation exercise

In addition to the literature review, we intend to consult with relevant stakeholders and experts as we summarize the results of our scoping review. This consultation exercise will enable us to identify potentially elusive domains of inquiry in the literature and gaps in knowledge using an iterative process. We will seek verbal or written input through consultation inquires with the American Mindfulness Research Association, the Centre for Mindfulness Studies, Mindful Schools, the Mind and Life Institute, and the International Association for Child and Adolescent Psychiatry and Allied Professions.

## Discussion

Our scoping review will provide a comprehensive overview of the existing evidence of MBIs for children and adolescents. The proposed framework for mapping the existing literature relating to pediatric mindfulness interventions includes a rigorous and transparent method, documented in such a way to promote ease of duplication and updates [21]. The identification of gaps in the current evidence will guide further research to enable the development of a more solid evidence base for children and adolescent MBIs. In particular, it is important to document the degree to which efficacious interventions initially designed for adults have been adequately extended and adapted for use with children and adolescents [2, 8].

We expect the findings of this scoping review to be of interest to a number of audiences, including the scientific community, practitioners, professional societies, policy-making organizations, and the lay public. Given the breadth of interest, we will present the results in a range of accessible formats, such as brief reports, presentations at academic conferences, press releases, and web postings on relevant websites [27]. In keeping with our primary objective, we believe that our study will enlighten practitioners and researchers working with MBIs, by providing a comprehensive view of the existing evidence of mindfulness interventions used in children and adolescents.

Furthermore, we expect to identify a number of studies involving children or adolescent MBIs reporting on a range of mental and physical health, cognitive, psychosocial, and affective outcomes in institutional and non-institutional settings. We therefore need to identify and understand the breadth of the literature based on MBI use in children and adolescents. We also anticipate that MBIs will appear promising for children and adolescent populations, although methodologically sound and higher quality evidence (e.g., better study designs, study evaluation methods, more objective measures, and larger sample sizes) research may be needed [2, 4–11, 14, 15].

Our hope is that this scoping review will not only inform future mindfulness research and applications of pediatric MBIs, but will also contribute to the evidence base and the improvement of age-appropriate mindfulness interventions, pediatric patient care, and well-being, during this time of exponential growth in the field of mindfulness.

## Supplementary Information

**Additional file 1.** Preferred Reporting Items for Systematic reviews and Meta-Analyses extension for Scoping Reviews (PRISMA-ScR) Checklist.

## Data Availability

Not applicable.

## Abbreviations

MBIs: Mindfulness-based interventions
PROSPERO: International Prospective Register of Systematic Reviews
AMRA: American Mindfulness Research Association
MBSR: Mindfulness-based stress reduction
RCTs: Randomized controlled trials
PCC: Participants–Concept–Context
JBI: Joanna Briggs Institute
CINAHL: Cumulative Index of Nursing and Allied Health Literature
Embase: Excerpta Medica database
ERIC: Educational Resources Information Center
MEDLINE: Medical Literature Analysis and Retrieval System Online
AMED: Allied and Complementary Medicine Database
MeSH: Medical Subject Headings
PRISMA-ScR: Preferred Reporting Items for Systematic reviews and Meta-Analyses extension for Scoping Reviews
ICF: International Classification of Functioning, Disability, and Health
